# A Novel CD48-Based Analysis of Sepsis-Induced Mouse Myeloid-Derived Suppressor Cell Compartments

**DOI:** 10.1155/2017/7521701

**Published:** 2017-02-26

**Authors:** Bei Jia, Chenchen Zhao, Guoli Li, Yaxian Kong, Yaluan Ma, Qiuping Wang, Beibei Wang, Hui Zeng

**Affiliations:** ^1^Institute of Infectious Diseases, Beijing Ditan Hospital, Capital Medical University, Beijing 100015, China; ^2^Beijing Key Laboratory of Emerging Infectious Diseases, Beijing 100015, China; ^3^Lab for Molecular Biology, Institute of Basic Theory on Chinese Medicine, China Academy of Chinese Medical Sciences, Beijing, China; ^4^Clinical Laboratory, Beijing Tongren Hospital, Capital Medical University, Beijing 100730, China

## Abstract

Myeloid-derived suppressor cells (MDSCs) are a heterogeneous subset of cells that expands dramatically in many disease states and can suppress T-cell responses. MDSCs mainly include monocytic and granulocytic subpopulations that can be distinguished in mice by the expression of Ly6G and Ly6C cell surface markers. This identification system has been validated in experimental tumor models, but not in models of inflammation-associated conditions such as sepsis. We challenged growth factor independent 1 transcription repressor green fluorescent protein (Gfi1:GFP) knock-in reporter mice with cecal ligation and puncture surgery and found that CD11b^+^Ly6G^low^Ly6C^high^ MDSCs in this sepsis model comprised both monocytic and granulocytic MDSCs. The evidence that conventional Ly6G/Ly6C marker analysis may not be suited to study of inflammation-induced MDSCs led to the development of a novel strategy of distinguishing granulocytic MDSCs from monocytic MDSCs in septic mice by expression of CD48. Application of this novel model should help achieve a more accurate understanding of the inflammation-induced MDSC activity.

## 1. Introduction

Myeloid-derived suppressor cells (MDSCs) are a heterogeneous subset of immune system cells capable of suppressing T-cell responses by upregulating the expression of arginase-1 (Arg-1), inducible nitric oxide synthetase (iNOS/NOS2), and reactive oxygen or nitrogen species [1–3]. In mice, MDSCs are firstly defined as CD11b positive and granulocyte antigen-1 positive (CD11b^+^Gr-1^+^) myeloid cells in cancer-related inflammation [4]. Since anti-Gr-1 antibody RB6-8C5 reacts with both lymphocyte antigens 6G and 6C (Ly6G and Ly6C) [5] and monocytes express Ly6C but not Ly6G, MDSCs are further distinguished as monocytic MDSCs (M-MDSCs, CD11b^+^Ly6G^low^Ly6C^high^) and granulocytic MDSCs (G-MDSCs, CD11b^+^Ly6G^high^Ly6C^low^) in experimental murine models [6–8].

MDSC expansion occurs in a number of pathological conditions, including malignancies [9], acute or chronic inflammation [10, 11], trauma [12], autoimmune diseases [13, 14], and organ transplantation [15]. Unfortunately, Ly6G and Ly6C expression by myeloid cells appears to be variable. It has been reported that expression of Ly6C is influenced by inflammatory stimuli, such as interferon-*γ* and tumor necrosis factor (TNF-*α*) [16–18]. Ly6G expression by cells with a mononuclear morphology has also been demonstrated during virus infection [19]. Sepsis can trigger production of many inflammatory cytokines along with strong stimulation of myelopoiesis in bone marrow and spleen. It is not clear whether the conventional interpretation of CD11b and Ly6G/Ly6C expression is suitable for distinguishing monocytic and granulocytic MDSCs in septic mice.

Growth factor independence 1 (Gfi1) is a nuclear zinc-finger protein that regulates the survival, proliferation, and differentiation of hematopoietic cells [20–22]. We previously reported that it was expressed in granulocytes and not in monocytes in the bone marrow [23–25]. We generated Gfi1:green fluorescent protein (Gfi1:GFP) knock-in reporter mice and used GFP expression to identify Gfil expression in granulocytes and monocytes in the mice under physiological conditions [26]. Gene expression array analysis revealed that expression of CD48, a glypiated-linked protein in the signaling lymphocyte activation molecule family, was inversely correlated with Gfi1 expression. CD48 was strongly expressed on monocytes and weakly expressed on granulocytes in healthy Gfil:GFP mice [23].

In this study, we challenged Gfi1:GFP knock-in reporter mice by cecal ligation and puncture (CLP) surgery and found that the population of CD11b^+^Ly6G^low^Ly6C^high^ cells in this sepsis model were heterogeneous and consisted of both monocytic and granulocytic MDSCs and that CD48 can distinguish monocytic and granulocytic MDSCs during infection.

## 2. Materials and Methods

### 2.1. Mice

Gfi1:GFP knock-in mice that were generated as described before [26] were kindly provided by Professor Tarik Möröy (Institut de Recherches Cliniques de Montréal, Canada). Male C57/BL6J specific-pathogen-free (SPF) mice of 6–8 weeks of age and weighing 18–20 g were purchased from the Institute of Laboratory Animal Science, Chinese Academy of Medical Science (Beijing, China). The mice were housed in groups of five with SPF soft wood shavings and ad libitum access to double-distilled water and commercial SPF pelleted food (GB14924.3-2010, HFK Bioscience, Beijing, China). All procedures performed on animals were approved by the Animal Care Research Ethics Committee of the Capital Medical University, Beijing, China.

### 2.2. Mouse Sepsis Model

Sepsis was induced by CLP or intraperitoneal injection of LPS. For CLP, Gfi1:GFP knock-in mice were anesthetized by intraperitoneal injection of ketamine 100 mg/kg and xylazine, 0.1 ml/10 g. Midline laparotomy was performed after skin disinfection with iodine tincture (2%). The cecum was divided approximately in half by a ligation distal to the ileocecal valve and was punctured once with an 18-gauge needle. The abdominal wall and skin were then sutured in layers with 4-0 silk, and 1 ml normal saline was injected subcutaneously for fluid resuscitation. This procedure induced sepsis with ~60% mortality over 13 days. To induce acute sepsis, Gfi1:GFP knock-in mice were given a single intraperitoneal injection of* Escherichia coli* pure LPS (Sigma-Aldrich, St. Louis, MO, USA) 10 mg/kg body weight.

### 2.3. Cell Preparation

Mice were sacrificed at 1, 7, and 12 days after CLP surgery or 3 hours after LPS injection. Blood, bone marrow, and spleen samples were collected aseptically from mice under deep anesthesia for subsequent experiments. Approximately 1 ml blood was collected into ethylenediaminetetraacetic acid (EDTA) coated tubes via retrobulbar vein puncture. Bone marrow cells were flushed from the femurs and tibias with phosphate buffered saline (PBS, Gibco, Thermo Fisher Scientific, Waltham, MA, USA) after bilateral hind limb dissection. The spleen was removed, minced with scissors, and ground using a 70 *μ*m cell strainer (BD Falcon, Bedford, MA, USA) under aseptic conditions. Erythrocytes were lysed with 1x Pharm Lyse solution (BD Biosciences, Sparks, MD, USA), and single spleen-cell suspensions were prepared by multiple pipetting and filtering through a 70 *μ*m nylon filter.

### 2.4. Flow Cytometry Analysis

Cells were labeled with anti-CD11b conjugated to PerCP-Cy5.5, anti-Gr-1 conjugated to phycoerythrin (PE, BD Biosciences, Franklin Lakes, NJ, USA), anti-Ly6C and anti-CD48 conjugated to allophycocyanin (APC, Affymetrix, eBioscience, Santa Clara, CA, USA), and anti-Ly6G conjugated to PE (BioLegend, San Diego, CA, USA). An isotype control antibody was used in each staining procedure. Single cell suspensions were prepared from bone marrow, blood, and spleen of Gfi1:GFP knock-in naïve controls, LPS-treated mice, and CLP mice. Cells were stained for 15 minutes at 4°C with primary antibodies in staining buffer (PBS with 2 mM EDTA and 0.5% BSA v/w). Flow cytometry assays were performed using a FACS-Calibur and Aria II flow cytometer (BD Biosciences, Franklin Lakes, NJ, USA). About 100,000 cells were analyzed with FlowJo 10.0 software (FlowJo, LLC., Ashland, OR, USA).

### 2.5. Cell Sorting

Cell sorting was performed with an Aria II flow cytometer with >95% purity. Bone marrow, spleen, and blood cells from Gfi1:GFP knock-in mice were stained with the anti-CD11b, CD48 and Gr-1 antibodies as described above and were defined as CD11b^+^Gr-1^+^CD48^−^ granulocytic subset and CD11b^+^Gr-1^+^CD48^+^ monocytic subset. The anti-CD11b, anti-Ly6G, and anti-Ly6C antibodies were used to sort CD11b^+^Ly6G^high^Ly6C^low^ granulocytic subset and CD11b^+^Ly6G^low^Ly6C^high^ monocytic subset. Sorted cells were washed and resuspended in sterile PBS. For CD4^+^ T cells sorting, cells were harvested from the spleen of C57/BL6J wild-type mice, and CD4^+^ T cells were purified by negative selection using a CD4^+^ T-Cell Isolation Kit II for magnetically assisted cell sorting (Miltenyi Biotec, Auburn, CA, USA) following the manufacturer's instructions.

### 2.6. Wright–Giemsa Staining and Cytological Analysis

Thin-layer preparations of suspensions containing 5 × 10^4^ cells in 300 *μ*l PBS were made at 500 rpm for 5 minutes using a cytospin-4 cytocentrifuge (Thermo Fisher). Cells were stained with a Wright–Giemsa kit (BASO, Wuhan, China) following the manufacturer's instructions. Briefly, cells were stained with buffer A for 1 minute and then buffer B for 10 minutes. After fixation and staining, slides were gently rinsed in tap water for 60 seconds and air-dried. A hematopathologist blinded to the study protocol conducted an independent analysis of the slides.

### 2.7. Cell Culture and Treatment

Following sorting, MDSC subgroups were cultured in RPMI 1640 medium supplemented with 10% FBS (R10, Gibco, Thermo Fisher Scientific). For in vitro LPS stimulation, 1 × 10^6^ cells were cultured in R10 with 100 ng/ml LPS for 3 hours and then assayed by flow cytometry.

### 2.8. T-Cell Proliferation Assay

Purified CD4^+^ T lymphocytes from naïve mouse spleens were labeled with 1 mM carboxyfluorescein N-hydroxysuccinimidyl ester (CFSE; Invitrogen, Portland, OR, USA). Cultures of 1 × 10^5^ labeled CD4^+^ T cells and unlabeled G-MDSC or M-MDSC cells with MDSC/T ratios of 1/4, 1/2, 1/1, and 2/1 were cultured in R10 in anti-CD3e (5 *μ*g/ml; functional grade, clone 145-2C11; eBioscience, Santa Clara, CA, USA) coated plates in the presence of soluble anti-CD28 (5 *μ*g/ml; functional grade, clone 37.51; eBioscience). After 60 hours, cells were collected and stained with APC-conjugated anti-CD4 antibody and 7-aminoactinomycin D (7-AAD, eBioscience). Proliferation of CFSE-labeled cells was assayed by flow cytometry.

### 2.9. Quantitative Real-Time PCR

Total RNA was extracted from sorted bone marrow and spleen MDSC subsets using TRIZOL reagent (Ambion, Thermo Fisher Scientific, Waltham, MA, USA). Approximately 20 ng RNA was transcribed into cDNA with a high capacity cDNA Reverse Transcription Kit (Applied Biosystems, Thermo Fisher Scientific, Foster City, CA, USA) following the manufacturer's protocol. Taqman master mix and probes for* Arg-1* (Mm00475988_m1),* Nos2* (Mn00440502_m1), and* Gapdh* (Mm03302249_g1) were purchased from Applied Biosystems. We used ABI Prism 7500 Sequence Detection System (Applied Biosystems, Foster City, CA, USA) to assay gene expression. Real-time PCRs were performed in triplicate for each sample, and averaged C_t_ values were used for calculations. Relative expression of Arg-1 and NOS2 mRNA was normalized to the bone marrow derived granulocytes from naïve mice.

### 2.10. Statistical Analysis

Results were reported as means ± SEM, and between-group differences were analyzed with unpaired-sample student *t*-tests. The significance of within-group differences was tested by analysis of variance. *P* < 0.05 was considered significant. GraphPad Prism 6.0c (La Jolla, CA, USA) was used for statistical analysis and for graphing data.

## 3. Results

### 3.1. Ly6G and Ly6C Expression in MDSC Subsets of Septic Mice

**Figure 1:**
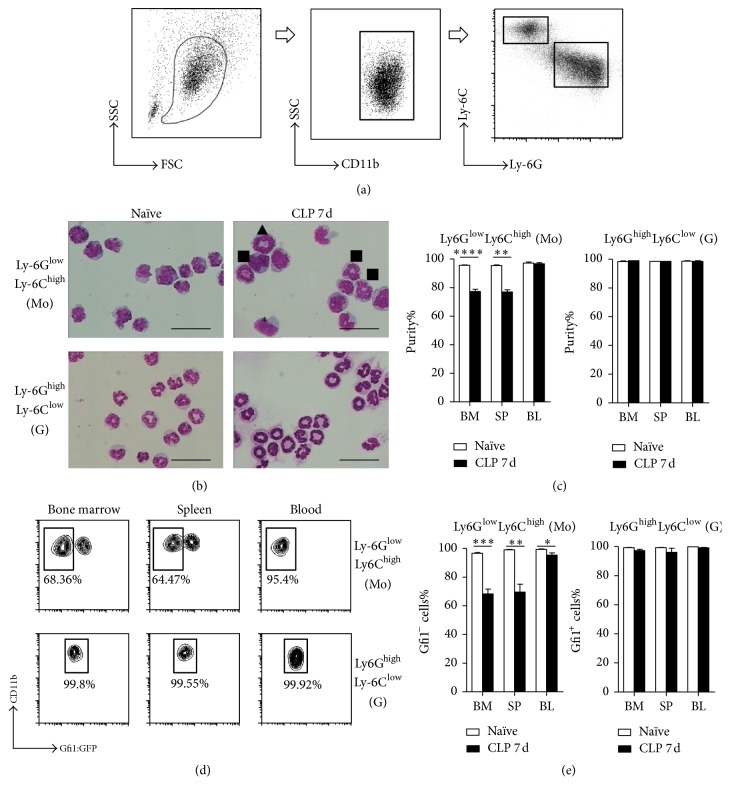
Purity of granulocytic and monocytic subpopulations identified by Ly6G and Ly6C. (a) Gating of monocytic and granulocytic MDSCs by Ly6C and Ly6G expression level. Cells were obtained from bone marrow, spleen, and blood from naïve and 7-day CLP Gfi1:GFP knock-in mice. MDSCs were gated out from CD11b^+^ cells and sorted as Ly6G^high^Ly6C^low^ granulocytes and Ly6G^low^Ly6C^high^ monocytes. (b) Representative photomicrographs (images of bone marrow are shown) of Wright–Giemsa stained MDSC subsets; 200 cells were counted in each cell preparation. Bar = 10 *μ*m, ^*∗*^monocyte, ^■^segmented granulocyte, ^▲^ring granulocyte. (c) Analysis of MDSC subset purity by cell morphology. (d) Gfi1:GFP fluorescence of each MDSC subset was read. Representative graphs of flow cytometry analysis are shown. (e) Purity analysis of MDSC subsets by FACS. Data are mean ± SEM of 5–8 mice per group, ^*∗*^*P* < 0.05, ^*∗∗*^*P* < 0.01, ^*∗∗∗*^*P* < 0.001, ^*∗∗∗∗*^*P* < 0.0001.

We assayed Ly6G and Ly6C expression in different MDSC subsets obtained from Gfi1:GFP knock-in mice with sepsis following CLP challenge. CD11b^+^ myeloid cell populations included Ly6G^high^Ly6C^low^ and Ly6G^low^Ly6C^high^ subpopulations by day 7 after CLP (Figure 1(a)). We sorted Ly6G^high^Ly6C^low^ and Ly6G^low^Ly6C^high^ cells from both naïve and CLP 7 d mice for Wright–Giemsa staining. Consistent with previous studies, the Ly6G^high^Ly6C^low^ myeloid cells of naïve mice had typical granulocytic morphology with ring-shaped or segmented nuclei, and the Ly6G^low^Ly6C^high^ myeloid cells had typical monocytic morphology (Figures 1(b) and 1(c)). The Ly6G^high^Ly6C^low^ myeloid cells of CLP mice were a homogeneous granulocyte population. The significant finding was that the Ly6G^low^Ly6C^high^ myeloid cells of CLP mice included populations with both granulocytic and monocytic morphology (Figures 1(b) and 1(c)).

The GFP expression of Ly6G^high^Ly6C^low^ and Ly6G^low^Ly6C^high^ myeloid cells confirmed the cell sorting and Wright–Giemsa staining results. More than 99.5% of the Ly6G^high^Ly6C^low^ cells from naïve mice were GFP^+^, whereas the Ly6G^low^Ly6C^high^ cells were GFP^−/low^, which indicated that they were granulocytes and monocytes, respectively (data not shown). Similar to Ly6G^high^Ly6C^low^ cells of naïve mice, the Ly6G^high^Ly6C^low^ cells of day 7 CLP mice were GFP^+^ (Figure 1(d)). In line with the morphologic data, CD11b^+^Ly6G^low^Ly6C^high^ cells included both GFP^+^ and GFP^−^ cells. The percentages of GFP^−^ cells were 68.36 ± 3.306% in bone marrow, 64.47 ± 2.563% in spleen, and 95.4 ± 1.453% in peripheral blood (Figures 1(d) and 1(e)). The results thus showed that CD11b^+^Ly6G^low^Ly6C^high^ cells of CLP mice included both granulocytes and monocytes.

**Figure 2:**
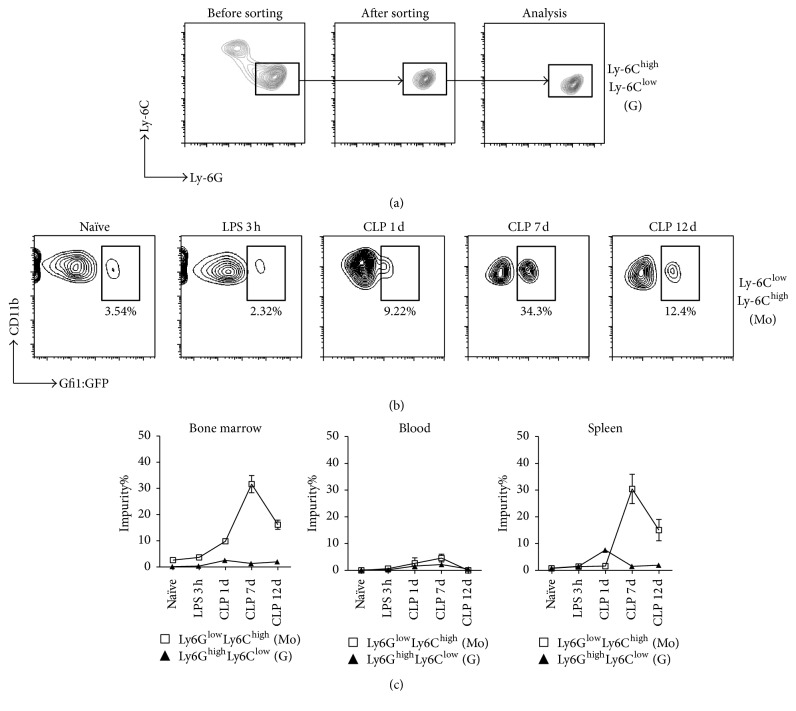
Influence of inflammation on Ly6C and Ly6G expression in MDSC subsets. (a) Sorted CD11b^+^Ly6G^high^Ly6C^low^ G-MDSCs from the bone marrow of wild-type mice were cultured with LPS (100 ng/ml) in vitro for 3 h, collected, and assayed by FACS. Representative graphs of flow cytometry analysis are shown. (b) Gfi1:GFP knock-in mice were exposed to LPS (i.p. 10 mg/kg) or CLP surgery. Bone marrow, spleen, and blood cells were stained with fluorescence-conjugated antibodies after 3 h LPS and on days 1, 7, and 12 after CLP. M-MDSCs were gated as CD11b^+^Ly6G^low^Ly6C^high^ and their purity was evaluated by Gfi1:GFP expression (graphs of bone marrow are shown). (c) Statistical analysis of flow cytometry data. Data are mean ± SEM of 5–8 mice per group.

The influence of inflammation on the expression of Ly6C and Ly6G by granulocytic MDSC was evaluated in CD11b^+^Ly6G^low^Ly6C^high^ cells following sorting and stimulation by lipopolysaccharide (LPS, 100 ng/ml) in vitro for 3 hours. We did not observe the emergence of the Ly6G^low^Ly6C^high^ cells during culture (Figure 2(a)), which implies that short-term inflammatory stimulation did not influence the expression of Ly6C and Ly6G by the granulocytes. We also investigated the dynamic changes in the purity of Ly6G^high^Ly6C^low^ and Ly6G^low^Ly6C^high^ myeloid cells at different stage of sepsis. As shown in Figures 2(b) and 2(c), naïve mice and LPS-treated mice had comparable proportions of Gfi1:GFP^+^ granulocytes within the bone marrow Ly6G^low^Ly6C^high^ population (3.654 ± 0.948% versus 2.603 ± 0.376%, *P* = 0.2563). However, the percentage of Gfi1:GFP^+^ in the Ly6G^low^Ly6C^high^ subpopulation significantly increased from day 1 after CLP, peaked on day 7 (31.64 ± 3.306%, *P* < 0.0001), and then gradually decreased to about 12% on day 12 (*P* < 0.0001). The same dynamic pattern was confirmed in the spleen cells of septic mice. Thus, Ly6G/Ly6C expression is not suitable for distinguishing granulocytes and monocytes in septic mice.

### 3.2. CD48 Labeling Distinguished Granulocytes from Monocytes in Septic Mice

**Figure 3:**
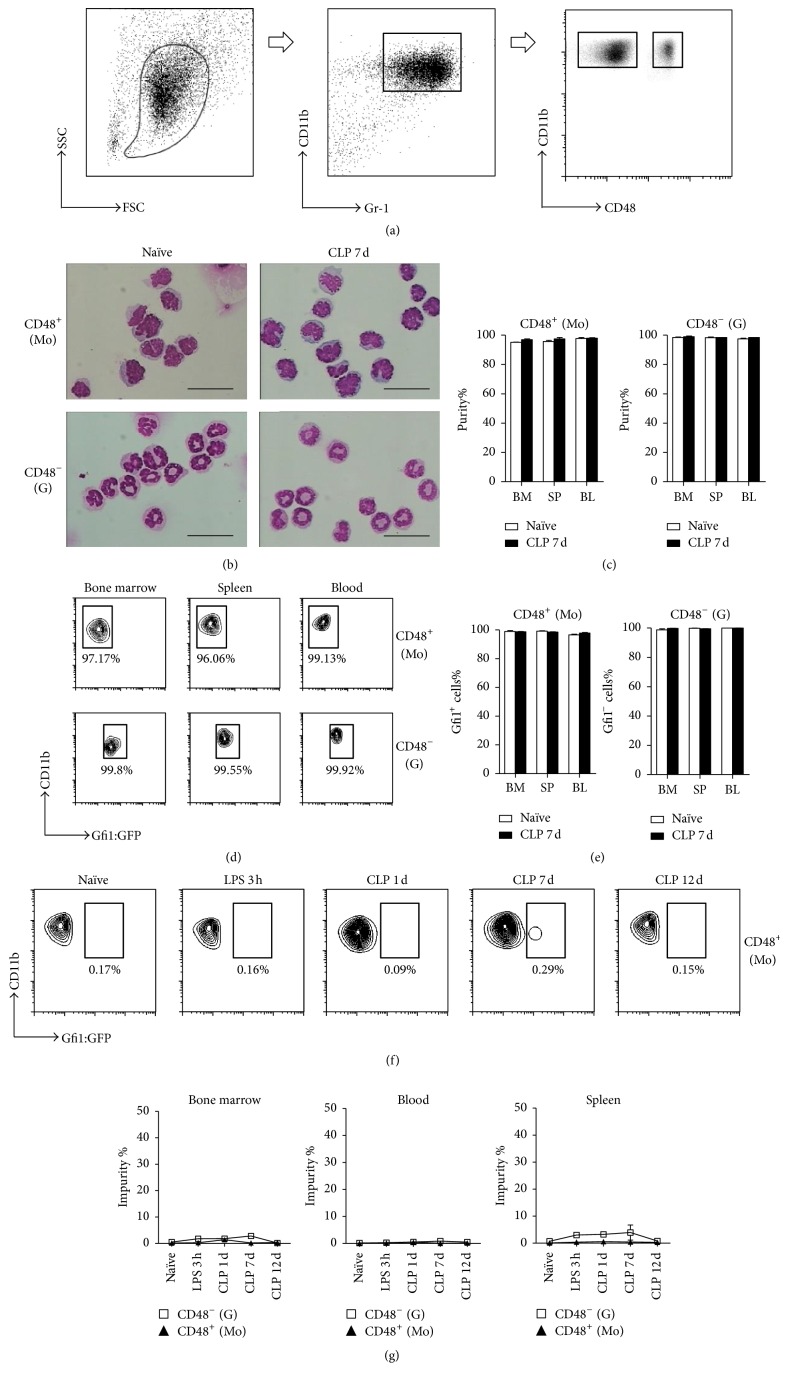
Granulocytic and monocytic MDSC subsets identified by CD48 expression. (a) Bone marrow, spleen, and blood cells were collected from naïve and 7-day CLP Gfi1:GFP knock-in mice. MDSCs were gated as CD11b^+^Gr-1^+^ and sorted by CD48 expression. G-MDSCs were identified as CD11b^+^Gr-1^+^CD48^−^ and M-MDSCs were identified as CD11b^+^Gr-1^+^CD48^+^ cells. (b) Representative photomicrographs (images of bone marrow are shown) of Wright–Giemsa stained MDSC subsets, Bar = 10 *μ*m. (c) Purity analysis of each MDSC subset by cell morphology. (d) Gfi1:GFP expression in CD11b^+^Gr-1^+^CD48^−^ and CD11b^+^Gr-1^+^CD48^+^ cells. Representative flow cytometry data are shown. (e) Purity analysis of MDSC subsets by Gfi1 expression. (f) Gfi1:GFP knock-in mice were treated with LPS (i.p. 10 mg/kg) or by CLP surgery. Bone marrow, splenic, and blood cells from 3 h LPS treatment and days 1, 7, and 12 CLP surgery were assayed. The purity of CD11b^+^Gr-1^+^CD48^−^ M-MDSCs was evaluated by Gfi1 expression. Representative graphs of bone marrow flow cytometry assay are shown. (g) Statistical analysis of flow cytometry data. Data are mean ± SEM of 5–8 mice per group.

Although granulocytes and monocytes can be distinguished in Gfi1:GFP knock-in mice in this mouse sepsis model, it would be more helpful to be able to distinguish them in wild-type mice. We thus used CD48 staining to assay the MDSC subsets in our sepsis model (Figure 3(a)). Fluorescence-activated cell sorting (FACS) analysis showed that, on day 7 after CLP, more than 99% of the CD11b^+^Gr-1^+^CD48^+^ cells from Gfi1:GFP knock-in mice were GFP^−^, and the CD11b^+^Gr-1^+^CD48^−^ cells were GFP^+^ (Figures 3(b) and 3(c)). Cell morphology analysis also confirmed that these two populations were monocytes and granulocytes, respectively (Figures 3(d) and 3(e)). Furthermore, we investigated CD48 expressions on CD11b^+^Ly6G^low^Ly6C^high^ and CD11b^+^Ly6G^high^Ly6C^low^ subsets, respectively. Consistent with the results shown in Figures 1(d) and 1(e), more than 99% of Ly6G^high^Ly6C^low^ myeloid cells in CLP 7 d mice were CD11b^+^CD48^−^, while CD11b^+^Ly6G^low^Ly6C^high^ monocytic MDSC contained both CD11b^+^CD48^+^ and CD11b^+^CD48^−^ populations (supplementary Figure 1 in Supplementary Material available online at https://doi.org/10.1155/2017/7521701).

We harvested bone marrow, spleen, and peripheral blood cells from Gfi1:GFP knock-in mice at 3 h, 1 day, 7 days, and 12 days after CLP for FACS analysis. The populations of CD11b^+^CD48^+^ cells were consistently GFP^−/low^ at each of the test intervals, whereas the CD11b^+^CD48^−^ populations were consistently GFP^+^ (Figures 3(f) and 3(g)). The same dynamic pattern was observed in the peripheral blood and spleens of septic mice. Thus, CD48 can distinguish granulocytes from monocytes in septic mice.

### 3.3. MDSC Purified by CD48-Based Cell Sorting Possessed Immune Suppressive Activity

**Figure 4:**
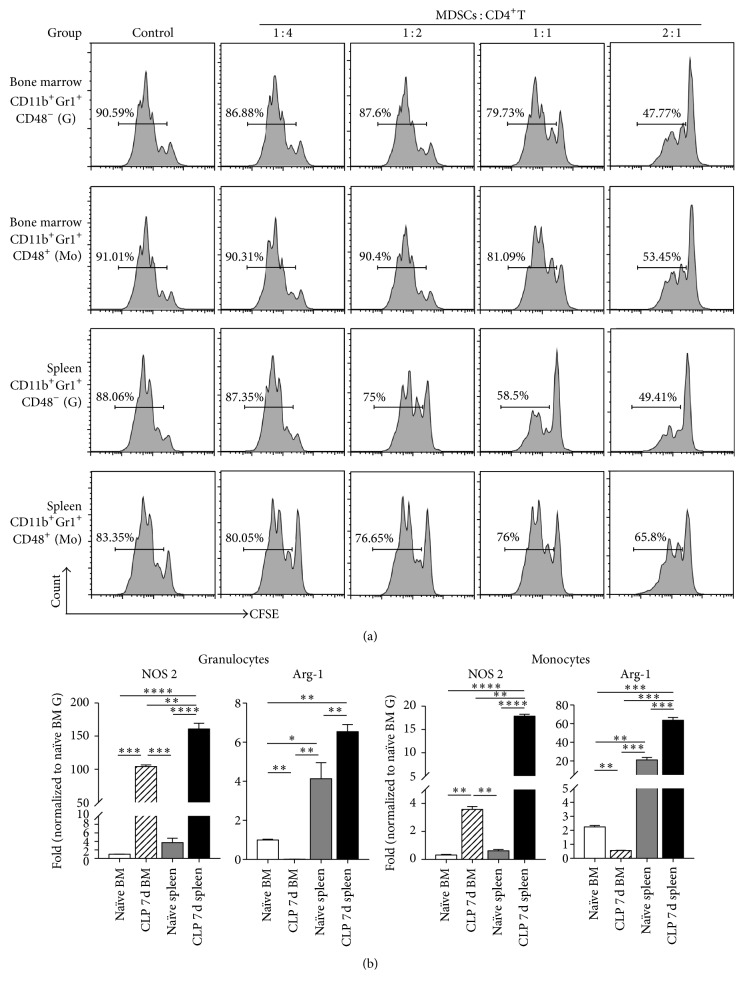
Immune suppression by MDSCs sorted by Gfi1:GFP expression. (a) Sorted MDSC subsets were cocultured with CFSE-labeled splenic CD4^+^ T cells isolated from naïve spleen by magnetic cell sorting. Anti-CD3 and anti-CD28 antibodies were applied to induce CD4^+^ T-cell proliferation. After 72 h, the proportion of proliferating CD4^+^ T cells was evaluated by CFSE staining and flow cytometry. Representative graphs show data from three independent replications. (b) Sorted CD11b^+^Gr-1^+^CD48^−^ G-MDSCs and CD11b^+^Gr-1^+^CD48^+^ M-MDSCs were analyzed by real-time qPCR. Probes detecting* NOS2* and* Arg-1* were used to quantify mRNA levels. Relative expression was normalized to bone marrow derived granulocytes from naïve mice. Data are mean ± SEM of 6–10 mice per group. The experiment was repeated three times, and the values presented are from one representative experiment. ^*∗*^*P* < 0.05, ^*∗∗*^*P* < 0.01, ^*∗∗∗*^*P* < 0.001, ^*∗∗∗∗*^*P* < 0.0001.

We further tested the immune suppressive abilities of monocyte and granulocyte subsets obtained by CD48-based cell sorting. CD11b^+^Gr-1^+^CD48^−^ granulocytes and CD11b^+^Gr-1^+^CD48^+^ monocytes from the bone marrow and spleen of mice on day 7 after CLP were cocultured with splenic CD4^+^ T cells from naïve mice. As shown in Figure 4(a), the percentage of proliferating splenic CD4^+^ T cells decreased significantly along with an increase in MDSC/T ratio. We assayed the mRNA expression of two enzymes, Arg-1 and NOS2, which regulated MDSC-mediated immune suppression in granulocytes and monocytes defined by CD48 analyzing strategy. Both G-MDSCs and M-MDSCs from the septic bone marrow and the spleens exhibited increased NOS2 mRNA levels. However, we observed a discrepancy of Arg-1 expression between bone marrow and splenic MDSCs. Bone marrow G-MDSCs and M-MDSCs showed decreased levels of Arg-1 mRNA, while splenic G-MDSCs and M-MDSCs displayed increased Arg-1 expression in septic mice (Figure 4(b)).

## 4. Discussion

Along with the development of sepsis, MDSC populations in the bone marrow, spleen, and blood expand and modulate host immune responses [27, 28]. MDSCs appear to be divided into monocytic and granulocytic subpopulations with distinct functions [8, 29]. Thus, the identification of specific MDSC subsets is prerequisite to a comprehensive understanding of their function. In malignancies and pathologies, the Ly6G and Ly6C cell surface markers can be used to distinguish CD11b^+^Ly6G^low^Ly6C^high^ monocytic MDSCs and CD11b^+^Ly6G^high^Ly6C^low^ granulocytic MDSCs [7, 30]. However, we found that, in sepsis, the Ly6G^low^Ly6C^high^ cell population included both monocytes and granulocytes, which indicated a variation of Ly6C and Ly6G expression in these myeloid cells under the inflammatory conditions of sepsis, and suggested that Ly6G/Ly6C expression may not be reliable for characterizing inflammation-induced MDSCs. Of note, similar phenomenon was also found in chronic inflammation [11]. Based on our previous report on transcription factor* Gfi1* and cell surface marker CD48 in murine myeloid hematopoiesis [23], we evaluated CD48 as a marker to effectively distinguish granulocytic MDSCs from monocytic MDSCs in septic mice.

We found that some G-MDSCs and M-MDSCs share the CD11b^+^Ly6G^low^Ly6C^high^ phenotype, leading to a mixed population of granulocytes and monocytes, both with the CD11b^+^Ly6G^low^Ly6C^high^ phenotype, and the potential of inaccurately identifying monocytes. This inflammation-related difference might be explained by the influence of inflammatory cytokines and mediators on the expression of myeloid cell surface markers [31]. That could explain the upregulation of Ly6C and downregulation of Ly6G molecules on the surface of granulocytes. However, the in vitro results of LPS stimulation imply that inflammation might not directly influence the expression of Ly6C and Ly6G by mature granulocytes. The significant increase in myelopoiesis in response to sepsis that we and others have identified occurs at the level of stem cells and primitive progenitors and may lead to dramatic changes in mature myeloid cells. The CD11b^+^Ly6G^low^Ly6C^high^ granulocytic subset that we observed may thus be a newly generated population arising during “emergency” hematopoiesis. The increase in proportion of CD11b^+^Ly6G^low^Ly6C^high^ granulocytes in the bone marrow and spleen accelerated with the progression of sepsis and with the progression of enhanced myelopoiesis. Thus, the emergency hematopoiesis during the development and aggravation of sepsis may contribute to the expansion of the novel granulocyte population.

Interestingly, we found that granulocytes with the Ly6G^low^Ly6C^high^ phenotype primarily resided in the bone marrow and the spleen, and not in the peripheral blood. This granulocyte subset might represent a stage of differentiation in the bone marrow and spleen, or a population with a reduced capacity of emigration from the hematopoietic organs. Another explanation is that the phenotype changes following migration into the peripheral blood. These possibilities should be investigated in future studies.

In contrast to dramatically decreased levels of Arg-1 in both G-MDSCs and M-MDSCs in the bone marrow, splenic G-MDSCs and M-MDSCs exhibited increased Arg-1 expression at day 7 after CLP surgery. Since inflammation-induced MDSCs arise from “emergency” hematopoiesis, such discrepancy of Arg-1 expression levels between bone marrow and splenic MDSCs might be a result from different inflammatory microenvironments for MDSC development during intramedullary hematopoiesis (the bone marrow) and extramedullary hematopoiesis (the spleen).

## 5. Conclusion

In summary, our findings present a novel characterization of myeloid cells during inflammation. In inflammatory conditions, CD48 staining and FACS allowed more accurate identification of bone marrow and splenic granulocytes and monocytes than possible with Ly6G/Ly6C. Using this method, we investigated the immune suppressive function of monocytic and granulocytic MDSCs. Our findings assist in studying myeloid cell subsets and add to our understanding of their immune regulatory functions.

## Supplementary Material

Supplementary Figure1: Evaluation of granulocytic and monocytic MDSCs purity identified by Ly6G/Ly6C and CD48 analyzing strategy. (a) Cells were obtained from bone marrow, spleen, and blood of naïve mice and day 7 CLP mice. MDSCs were gated out from CD11b^+^ cells and then gated as Ly6G^high^Ly6C^low^ granulocytes and Ly6G^low^Ly6C^high^ monocytes. The purity of these two subsets were further assessed according to CD48 expression. (b) Statistical analysis of flow cytometry data. Data are mean ± SEM of 6-10 mice per group and representative graphs are shown. ^**^P< 0.01, ^***^P< 0.001.
