# Supplementary figures and images for: A Novel CD48-Based Analysis of Sepsis-Induced Mouse Myeloid-Derived Suppressor Cell Compartments
Source: Mediators Inflamm. 2017 Feb 26;2017:7521701. doi: 10.1155/2017/7521701 (PMC5346402; doi:10.1155/2017/7521701)

Supplementary data

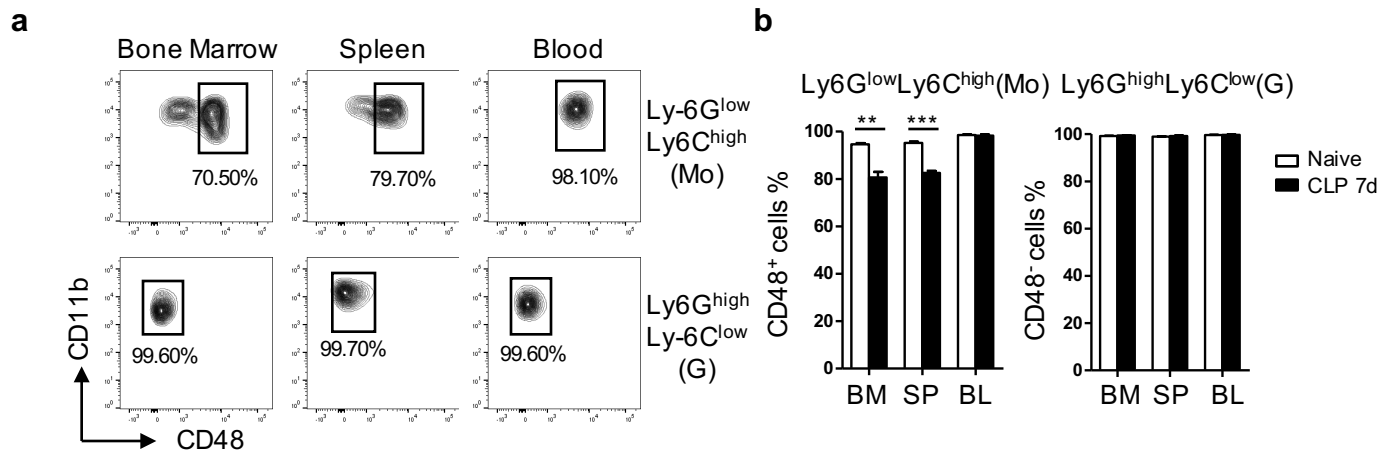

Jia et al  
Supplementary figure 1

Supplement: Supplementary file 1 — Supplementary Figure1: Evaluation of granulocytic and monocytic MDSCs purity identified by Ly6G/Ly6C and CD48 analyzing strategy. (a) Cells were obtained from bone marrow, spleen, and blood of naïve mice and day 7 CLP mice. MDSCs were gated out from CD11b+ cells and then gated as Ly6GhighLy6Clow granulocytes and Ly6GlowLy6Chigh monocytes. The purity of these two subsets were further assessed according to CD48 expression. (b) Statistical analysis of flow cytometry data. Data are mean ± SEM of 6-10 mice per group and representative graphs are shown. **P< 0.01, ***P< 0.001. [file 7521701.f1.pdf]
